# Patients to learn from: on the need for systematic integration of research and care in academic health care

**Published:** 2017-09-14

**Authors:** Martin Boeckhout, Philip Scheltens, Peggy Manders, Cees Smit, Annelien L Bredenoord, Gerhard A Zielhuis

**Affiliations:** ^1^ *BBMRI-NL, University Medical Centre Groningen, the Netherlands*; ^2^ *Julius Center for Health Sciences and Primary Care, Department of Medical Humanities, University Medical Center Utrecht, Utrecht, The Netherlands*; ^3^ *Alzheimer Center, VU University Medical Center, Amsterdam, the Netherlands*; ^4^ *Radboud Biobank, Radboud university medical center, Nijmegen, the Netherlands*; ^5^ *Department for Human Genetics, Radboud university medical center, Nijmegen, the Netherlands*; ^6^ *Department for Health Evidence, Radboud university medical center, Nijmegen, the Netherlands*; ^7^ *Patient advocate, VSOP, Soestdijk, the Netherlands*

**Keywords:** research-care integration, infrastructure, IT, Parelsnoer Institute

## Abstract

Patients suffering from rare, extreme or extremely complex sets of symptoms have something to expect from efforts to improve care through research. Biomedical research and care have often been approached as distinct worlds which are and should be only loosely connected. For observational research focusing on data drawn from real-world settings, however, that approach is found wanting. Integrating research and care responsibly is the main challenge instead. Integrated IT infrastructures facilitating Personalized medicine and Big Data are crucial components of a learning health care system, in which patients regularly play a double role: as individuals to be treated and as cases to learn from. Drawing on the example of the Dutch Parelsnoer Institute (PSI), a national biobanking and IT infrastructure integrated with clinical care procedures, this article outlines the reforms that are needed.

Systematic integration of research and care offers a promising avenue, provided that a number of conditions are met: data and IT infrastructures will require overhauls in order to facilitate secure, high-quality data integration between research and care; institutional focus is needed to bring patient populations and expertise together; ethical frameworks and approaches for integrating research and care responsibly require further elaboration; clinical procedures and professional responsibilities may need to be adapted in order to accommodate research requirements in clinical processes; and involvement of patients and other stakeholders in design and research priority setting is needed to further the goals of real-world and patient relevance.

**Relevance for patients:**

Integrating research and care in academic medicine in a more systematic fashion offers a promising perspective to current and future patients. In order to live up to these promises, research and care should be integrated more systematically in academic health science, with patients being included as research participants by default. Data and tissue infrastructures and facilities can provide a platform for doing so. At the same time, many issues remain to be settled. New ethical ways and means for protecting and respecting patient-participants in such a double role are also needed in this respect. In this way a deeper transformation is at stake as well: a change towards a setting in which patients fully take center stage in debate and action on the future of biomedicine.

## 1. Introduction

Biomedical research and care have often been approached as distinct, only loosely connected worlds. That approach has served patients, medicine and health care well in many ways. For instance, the approach has helped to protect patients from potential harms associated with participation while facilitating methodologically sound hypothesis-driven research. Currently, however, the approach is often found wanting. Moreover, research participants are only marginally subject to risks of physical burden and harm. Data-driven research efforts do not need to interfere with the provision of individual care, which was the prime ethical rationale for keeping health care and research apart. Research is turning to data-driven methodologies and approaches which draw on high-quality real-world data in health research collected in clinical settings. 

Patients play a double role in such approaches: as individuals to be treated and as cases to learn from [1,2]. Research focusing on patients’ ‘data doubles’ is associated with different risks and concerns about transparency, rights and informational harm. Tackling such risks does not call for a sharp separation as it does for responsible integration. Advanced data and IT infrastructures make such integration both necessary and possible. How can health care and research be organized in order to serve as a dual engine for treatment and scientific discovery? In our view, patients suffering from rare diseases, along with other ‘exceptional’ patients, merit particular attention when pursuing integration of research and care. Academic medicine, which often focuses on such patients, should take the lead in developing such integration. Drawing in particular on the Dutch example of the Parelsnoer Institute (PSI), this article outlines the reforms that we believe are needed

## 2. IT infrastructure, health care data and the integration of research and care: institutionalizing the connection between personalized medicine and the learning healthcare system

The integration of research and health care plays a central role in contemporary overarching models of biomedicine. In relation to Personalized or Precision Medicine, research and care are considered to be integrated at the level of (small groups of) patients. By adding an experimental dimension to diagnosis and treatment, data-driven innovations could help to differentiate therapeutic regimes to suit more specific patients and patient groups. In visions of learning health care systems, the integration of research and care is understood to be forged at a systemic level, by studying real-world patterns, drawing scientific insights and evidence from these and implementing these in health care practice. In such a model, data-driven innovations are more closely associated with the tools and research approaches of epidemiology. Both perspectives ultimately feed into one another: clinical decision-making relies on epidemiological evidence, while population-level insights can only emerge from carefully crafted standardized data collection efforts. That being said, good institional and infrastructural arrangements are required in order to enable the translation efforts between both perspectives on health and disease. Academic health care is a crucial site for achieving this, provided that academic health care settings are turned into local learning healthcare systems [1,3]. 

For integration of research and care into their mission and daily activities, institutions have to be equipped and organized in such a way that uncertainties of biomedical knowledge and interventions pertaining to their patients can be systematically explored. Clinical data, for one, should be collected in ways which allow for further exploration and integration into research databases. Biobanking infrastructure and advanced electronic health record systems collecting comprehensive clinical data and capable of catering to research and care simultaneously should stand at the heart of such integration [4]. These infrastructures can help to feed the discovery phase of translational research (for instance by facilitating the search for novel biomarkers), allowing for more systematic and far-reaching exploration of patient needs and how to meet them. Given the need for systematic data integration between research and care, meeting the highest informational privacy and data security standards in such infrastructures is a requisite

## 4. Example: the Parelsnoer Institute

Many initiatives in which aspects of research and care are integrated are ongoing. One prominent initiative in The Netherlands which could serve as a source of inspiration is the Parelsnoer Institute (PSI). PSI was established in 2007 by the Netherlands Federation of University Medical Centers (NFU) in order to improve diagnosis, prevention and treatment of complex disorders and to facilitate personalized medicine. Here we explain the aspects of PSI that are most relevant. More details are available in a recently published PSI marker paper as well as on the PSI website, where PSI protocols and model regulations are available for download [5,6]. 

PSI provides a national research infrastructure integrated with clinical care procedures, in which clinical researchers from all University Medical Centers (UMCs) collaborate and prospectively collect and store biomaterial such as DNA and serum and associated data from large cohorts of clinically documented patients. It develops and offers ready-to-use harmonized procedures in compliance with recognized national and international standards to ensure uniform collections. Standard operating procedures throughout the phases of the biobanking process are developed and implemented to ensure quality and uniformity of the collections.

Cohorts of disease-specific collections of biomaterials and data, the so-called Pearls, are collectively managed by clinician-researchers in multiple UMCs. Data in each Pearl is set up according to definitions, standards and procedures which are specified in information models drawn up by clinician-researchers according to international standards. The models are closely integrated into Electronic Health Records (EHRs) and routine care procedures, thus minimizing the registration burden.

**Table:** 

Parelsnoer Institute
Established in 2007
A collaboration involving 8 University Medical Centers and 15 clinical specialties
Prospective collection of biomaterials and data through shared clinical and data standards, information models and SOPs
Data and sample collection integrated into routine health care and electronic health record systems
Patients provide broad consent for use of their samples and data
Overview of available data and samples through https://catalogue.bbmri.nl For more information, see (5) or http://www.parelsnoer.org/page/en/

Patients may benefit directly from such integration. In particular, tailoring standard health care infrastructure to systematically feed into research requires ongoing harmonization of clinical care routines, thus minimizing burden to the patient and making optimal use of the data that are gathered throughout the care process. Moreover, by implementing research protocols, both patient and research quality could benefit. Such harmonization, and the ongoing process of tinkering and reflection on what data to collect in what ways, facilitates collective learning and stimulates the wider adoption of clinical best practice. In this way, patient care stands to become enhanced by research processes themselves – not just by the outcomes of research [7]. 

The experiences of clinical biobanking infrastructure for research into neurodegenerative diseases within PSI provide a case in point [8,9]. The Alzheimer Center of the VU Medical Center, a partner in PSI, holds a strict protocol for patients suspected from Alzheimer disease. During the standard diagnostic workup patients are seen by a medical neurologist, specialized nurses and a neuropsychologist. Team members collect visual and electronic read-outs of the brain (MRI, EEG), draw blood and conduct a lumbar puncture to collect cerebrospinal fluid (CSF). Processing and analysis of MRI images for research into prognostic markers and models for neurodegenerative disease will be automated in the near future, without radiologists having to manually assess and score these images. A standard set of neuropsychological tests is also part of the routine. 

The establishment of these novel routines and infrastructures has led to rapid improvements in patient care throughout participating institutions, by stimulating inter-institutional comparison and learning processes for all kind of aspects of clinical procedures, by driving the uptake and diffusion of clinical best practice, as well as by facilitating ongoing comparison and improvement of clinical outcomes. A tangible outcome of such improved care is evidenced by the fact that the diagnostic protocol for PSI is still widely used in most UMCs by participating clinicians [7,8].

## 4 Integrating care and research: aspects to consider

How might opportunities for learning through systematic integration of research and care be reinforced? A first point to consider pertains to the patients on which to focus. For a considerable number of patients, evidence-based standards and treatment options are hardly available [10]. This involves particularly patients suffering from rare disorders as well as patients suffering from (multiple) complex diseases and/or diseases which require complex treatment. In many cases, people suffering from rare diseases remain undiagnosed for a long time and need to go through considerable trajectories to receive a proper diagnosis [11]. For patients suffering from complex diseases, or from diseases requiring complex treatment, standard-level care will often also prove insufficient. 

A particularly close integration of research and care is warranted to improve these patients’ predicament. For such patients, receiving effective treatment or clarity on the underlying mechanisms involved in their disease is a puzzle, and providing professional care will often be a matter of trial and error. A full investigative picture of a clinical presentation will likely help patients to receive the best available healthcare. Moreover, these patients’ unmet needs are relevant to improve biomedicine: their symptoms might help to raise hypotheses for new studies. By enrolling these patients in clinical trials and other clinical studies, the evidence base could be improved on. Such patients add to the variability and hence to the probability to detect meaningful differences. Furthermore, patients with extreme, contrasting clinical features (such as a very poor or very favorable response to therapy) are likely to yield novel insights into and understanding of basic underlying pathological and biological mechanisms involved in health and disease [12,13]. Patients suffering from similar, less pronounced symptoms may also profit from these insights. 

Second, focusing in particular on such patients will also involve attending to the institutional focus of academic medicine. Academic health care centers should ideally focus on patients who cannot be treated sufficiently in ordinary clinical care procedures. For these patients in particular, research and care should go together and be designed and organized for combining duties of research and care. Sufficient expertise in specialist centers is a crucial prerequisite in this regard, as is the concentration of care [10]. In order to amass sufficient numbers of academic patients and to compare these against other patients suffering from comparable afflictions, such concentration of care and research capacities should also accommodate cross-institutional, national and ideally even transnational aggregation and exchange of data. 

Third, integrating research and care also implies attention to clinical procedures and professional responsibilities in order to accommodate research routinely. This involves practical changes, such as adding research nurses permanently to the staff and accommodating research requirements into clinical processes. Moreover, it involves closer partnerships with other academic hospitals and stakeholders in order to be able to conduct cutting-edge research. The collaborations set up through the Parelsnoer Institute are an example of this. During the first visits at the clinical departments, patients are asked to participate and consent to the collection and use through PSI of their clinical and follow-up data, images, residual tissue, as well as additional biomaterial of particular relevance to the understanding of their disease. As clinical care and clinical research are fully integrated in a natural and standardized way, patients do not have to make additional efforts, nor have to undergo extra burden while participating in research. 

PSI provides but one way of pursuing such integration. Patient registries can also facilitate overviews and comparisons of standards of care [14]. Moreover, prospective cohort studies such as the Prospective Dutch Colorectal Cancer cohort (PLCRC) may also serve as a model for a cohort infrastructure which can provide clinicians and researchers with baseline data and IT platforms which facilitate further studies and clinical trials [15]. For all of these initiatives, maximizing the accessibility and use of such infrastructures by governing them according to FAIR principles of data stewardship is crucial [16,17]. 

Fourth, principles and practical requirements of research ethics will also need to be updated [18]. The paradigm of participant protection stands at the heart of traditional research ethics. This paradigm is associated with risks of physical harm and the issue of therapeutic misconception, when patients unduly consider that research could have direct benefits for their individual predicament. The integration of research and care by data-driven raises different concerns. Risks of informational harm are more prominent in such situations, while research participation may now offer some promise for patients individually as well. Dealing with such concerns requires responsible integration of research and care instead of aiming for separation. 

The principle of privacy and data protection by design is particularly important in this regard [19]. In the PSI IT infrastructure, data protection was designed into the infrastructure by double encryption and deidentification of data and samples: first when periodically uploading data in encrypted fashion into a central database, and once more before making data and samples available to researchers. 

Ethics and oversight systems for a learning health care system are another point for discussion. Such oversight could entail a focus on protecting risks across the board without relying on unwarranted assumptions about inherent differences in risk between research and care [20]. The burden of integrating research into care for individual patient-participants should be minimized, with remaining risks and benefits regularly assessed through a combination of ethics review, governance and oversight [21]. Emerging guidelines and practices in the area of biobanking also point towards relevant developments [22]. 

Fifth, patients’ dual role as patients and research participants will also require more involvement and new forms of informed consent. Patient-participants should be adequately informed about the sense in which they may and may not stand to benefit about joining and being treated in a program for integrated research and care. This includes taking enough time and effort to inform, educate and discuss the kinds of care and research processes they will be and are participating in, enabling them to benefit from and keeping them up-to-date about current scientific state of the art and any relevant developments for them personally [23]. Instead of being project-specific, consent can be ‘broad’ by pertaining to research in a particular area or research program. A crucial component of such broad consent is that it involves consent for governance; consent, that is, to a particular way of managing resources and deciding on proper use [24,25]. Informed consent provided for PSI, a template of which is available on the PSI website, provides an example of this. Novel interfaces which can facilitate online interaction, such as those linked to concepts of dynamic consent, are promising tools in this regard [26,27]. 

Sixth, efforts to ensure that research agendas in biomedical research cater to urgent medical needs, are also needed. The need for real-world relevance of research in countering problems of research waste also merits a more systematic approach to involvement, monitoring and data collection of patients [28]. The experiences in the Parelsnoer Institute suggest that such infrastructures may at the same time provide a more efficient platform for translational research as well [7]. Moreover, involvement of patient advocates and organizations could become a standard feature of the research agenda-setting process, protocol design and execution. Given the right circumstances – well-read up patient advocates, sufficient and well-thought out organizational support – patient and public engagement can help to improve the relevance and quality of research tremendously [29,30]. 

In sum, systematic integration of research and care offers a promising avenue for exceptional patients, provided that a number of conditions are met: data and IT infrastructures will require overhauls in order to facilitate secure, high-quality data integration between research and care; institutional focus is needed to bring patient populations and expertise together; ethical frameworks and approaches for integrating research and care responsibly require further elaboration; clinical procedures and professional responsibilities may need to be adapted in order to accommodate research requirements in clinical processes; and involvement of patients and other stakeholders in design and research priority setting is needed to further the goals of real-world and patient relevance

## 5. Conclusion

Integrating research and care in academic medicine in a more systematic fashion offers a promising perspective to both current as well as future patients. In order to live up to these promises, research and care should be integrated more systematically in academic health science, with patients being included as research participants by default. Data and tissue infrastructures and facilities can provide a platform for doing so. At the same time, many issues remain to be settled in joint efforts of all involved, including clinicians, researchers, IT specialists, hospital boards, and patients. New ethical ways and means for protecting and respecting patient-participants in such a double role, such as through new forms of consent, more active attention to feedback of research findings, and more transparent governance arrangements, are also needed in this respect. In this way a deeper transformation is at stake as well: a change towards an environment in which patients fully take centre stage in debate and action on the future of biomedicine
